# Changes in Epithelial Cell Polarity and Adhesion Guide Human Endometrial Receptivity: How In Vitro Systems Help to Untangle Mechanistic Details

**DOI:** 10.3390/biom15081057

**Published:** 2025-07-22

**Authors:** Irmgard Classen-Linke, Volker U. Buck, Anna K. Sternberg, Matthias Kohlen, Liubov Izmaylova, Rudolf E. Leube

**Affiliations:** Institute of Molecular and Cellular Anatomy, RWTH Aachen University, Wendlingweg 2, 52074 Aachen, Germany; vbuck@ukaachen.de (V.U.B.); mkohlen@ukaachen.de (M.K.); lizmayolova@ukaachen.de (L.I.); rleube@ukaachen.de (R.E.L.)

**Keywords:** endometrial epithelium, polarity, cell–cell adhesion, implantation, receptivity, trophoblast invasion, epithelial–mesenchymal transition

## Abstract

Tissue remodeling of human endometrium occurs during the menstrual cycle to prepare for embryo adhesion and invasion. The ovarian steroid hormones 17β-estradiol and progesterone control the menstrual cycle to achieve the receptive state during the “window of implantation” (WOI). Here, we focus on the human endometrial epithelium and its changes in polarity, adhesion, cytoskeletal organization and the underlying extracellular matrix enabling embryo implantation. The adhesion and invasion of the trophoblast via the apical plasma membrane of epithelial cells is a unique cell biological process, which is coupled to partial epithelial–mesenchymal transition (EMT). Given the fundamental species differences during implantation, we restrict the review mainly to the human situation and focus on cell culture systems to study the interaction between human trophoblast and endometrial cells. We summarize current knowledge based on the relatively scarce in vivo data and the steadily growing in vitro observations using various cell culture systems.

## 1. Introduction

Human embryo implantation is a complex process which involves several molecular steps underlying attachment, adhesion and penetration of the embryo. Infertility affects up to 15% of reproductive-aged couples worldwide [1]. Based on studies in reproductive biology, physiologist Robert Edwards successfully pioneered conception through in vitro fertilization (IVF), which led to the birth of Louise Brown, the first IVF child in 1978. Together with the gynecologist Patrick Streptoe and embryologist Jean Purdy, he founded the first IVF program for infertile patients and trained other scientists in the relevant techniques. In 2010, Robert Edwards was awarded the Nobel Prize in Physiology or Medicine “for the development of in vitro fertilization” [2].

Although ~5 million children had been born by IVF already in 2013 [3] and technological refinements have increased pregnancy rates, implantation failure persists, and the underlying processes are not well understood. Besides poor embryo quality [4], implantation failure is the major limiting step in assisted reproductive technology (ART). It has been estimated that about 30% of pre-clinical losses are due to implantation failure [5]. Based on a study of the U.S. Centers for Disease Control and Prevention (CDC) in 2011, only 30% of single-embryo transfers resulted in pregnancy [6]. While the latest CDC study (https://www.cdc.gov/art/php/national-summary/index.html, accessed on 20 April 2025) reported that 37.5% of ART cycles result in live birth delivery of women with an average age of 36 years, it is still unknown why human reproduction is not more efficient.

Dynamic membrane reorganization and a change in luminal and glandular epithelial cell polarity occur during the “window of implantation” (WOI). If this process is interrupted, implantation cannot occur. Since the endometrial epithelium plays such an important role for successful implantation, we will focus on aspects of plasma membrane transformation (PMT) that contribute to embryo receptivity in vivo and its investigation in suitable cell culture systems.

## 2. Part I: In Vivo Observations

### 2.1 Dynamic Changes of the Endometrial Epithelium Occur During the Menstrual Cycle

**Figure 1:**
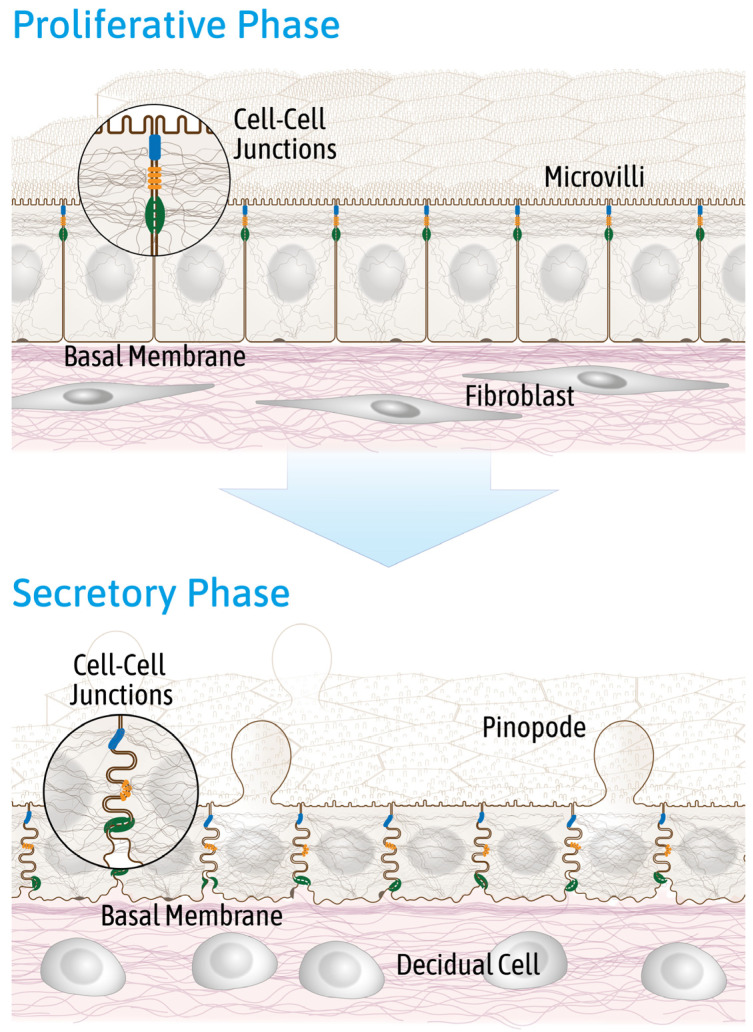
The schematic drawings depict the surface endometrial epithelial cell layer in the proliferative and the secretory phase of the menstrual cycle. In the proliferative phase, the endometrial epithelium is highly polarized. Columnar shaped epithelial cells contain apical microvilli and a typical subapical tripartite junctional complex. The underlying stroma consists of elongated fibroblasts embedded in extracellular matrix with a loose fibrillar meshwork. In the secretory phase, the cuboidal shaped epithelial cells become less polarized with reduced microvilli and form apical membrane protrusions (pinopodes). The cell–cell junctions disperse along the basolateral membrane. Stromal edema occurs at the mid-secretory phase. The stromal fibroblasts differentiate around day 24 into pre-decidual cells with epitheloid cell appearance producing extracellular matrix proteins. Figure modified from [23].

The endometrium lining the uterine cavity is divided into the permanent *stratum basale* and the temporary *stratum functionale*, which is shed during desquamation at the end of each menstrual cycle and grows back during the proliferative phase of the menstrual cycle. The luminal surface epithelium is the first contact site for adhesion and invasion of the developing blastocyst. In contrast to other simple epithelia, the human endometrial epithelium undergoes major morphological changes during the menstrual cycle in preparation for embryo implantation. During the proliferative phase large tubular glands are formed, which are embedded in the stromal *stratum functionale*. This phase of the menstrual cycle is under the control of estrogen and occurs between days 6 and 14. The epithelial cells present a polarized phenotype (Figure 1). Their apical membrane regions feature a typical brush border consisting of microvilli. The epithelial cells are connected by junctional complexes at their upper basolateral side, separating apical from basolateral domains. The junctional complexes consist of tight junctions at the top, followed by actin-anchoring adherens junctions and keratin filament-anchoring desmosomes below.

The endometrial stroma consists of extracellular matrix-producing fibroblasts, blood vessels and immunocompetent cells, i.e., large granular lymphocytes (LGLs) that increase in number during the secretory phase of the menstrual cycle [7,8,9].

The endometrial epithelial phenotype changes profoundly during the predominantly progesterone-driven secretory phase (days 15–28) to support embryo adhesion, implantation and growth (Figure 1). Glycogen-containing secretory vacuoles emerge in the glandular epithelium and are secreted for embryo nutrition. Specialized cellular apical protrusions (pinopodes) appear in the surface epithelium [10,11], reviewed by Quinn et al., 2020 [12]. They are rich in actin and may be involved in secretion and resorption. Cell–cell junctions get more evenly distributed along the basolateral membrane. In addition, the epithelial cell morphology changes from a columnar to a more cuboidal type [13,14,15].

Changes occur also in the extracellular matrix during the menstrual cycle as evidenced by immunofluorescence studies. In the interstitial matrix of decidualizing stroma type VI collagen was found to be abundant during the proliferative phase but was progressively lost during the secretory phase [16]. In addition, the subepithelial basement membrane composition changes during the menstrual cycle. Reduced expression of laminin during the mid to late secretory phase compared to the proliferative phase was especially significant in the surface epithelium. Type IV collagen was reduced during the late proliferative phase [17]. In a morphometric study using electron microscopy [18], the basement membrane of the luminal epithelium was thinnest at day 6 after the luteinizing hormone (LH) surge, i.e., at the time of implantation. The authors suggested that this change may facilitate embryo implantation.

The process of stromal decidualization is also an important predictive parameter for successful implantation after first adhesion and invasion of the embryo. In contrast to most mammals, pre-decidualization in the human endometrium is triggered by progesterone and c-AMP increase. It starts already around day 24 in the secretory phase and does not require embryo implantation. Morphologically, the elongated fibroblast-like stromal cells differentiate to epitheloid-like cells with round nuclei. In the increasing cytoplasm of the decidual cells, glycogen and lipid droplets accumulate, and hormones and extracellular matrix are produced. For more details regarding cyclic decidualization, we refer the reader to the comprehensive review of Gellersen and Brosens, 2014 [19].

**Figure 2:**
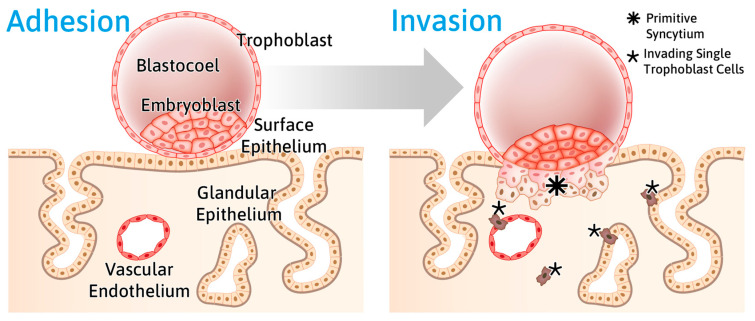
The scheme highlights the adhesion of the blastocyst to the endometrial surface epithelium and its subsequent invasion during the WOI (6–10 days after ovulation, 7–11 days after LH peak). After adhesion to the apical side of the endometrial epithelial cells, the trophectoderm forms a multinuclear syncytium, the syncytiotrophoblast, which transmigrates through the epithelial cell layer and invades the decidual stroma compartment to form the placenta. Single extravillous trophoblast cells migrate through the decidua and invade glands and spiral arteries from their basal sides for histiotrophic and hemotrophic nutrition. Figure reproduced from [23].

The embryo can only implant during the “phase of receptivity”, also designated as the “window of implantation” (WOI) [20,21]. In humans, the WOI is typically between days 20 and 24 of a regular 28-day cycle [22]. The blastocyst adheres to the apical side of the endometrial epithelium, which acts normally as a barrier (Figure 2).

### 2.2. Reduced Endometrial Epithelial Cell Polarity Favors Receptivity

The endometrium like all other simple epithelia is polarized. Cell polarity is defined as asymmetry in cell shape, protein distribution and cell function [24]. Polarized epithelial cells have two distinct plasma membrane domains, i.e., an apical domain which faces the lumen and a basolateral domain. The apical junctional complex, a structure which is conserved from nematodes to vertebrates, regulates the identities of both membrane domains [24]. It is located at the boundary between the apical and lateral membrane domains [25].

The Crumbs PALS1-PATJ complex and the PAR3-PAR6-aPKC complex [26] are localized at the tight junction seal in mammalian cells and determine apical domain formation. The Scribble complex, on the other hand, determines the basolateral domain [27]. Whitby et al., 2018 [28], observed a significant downregulation of apical Crumbs in the luminal endometrial epithelium during the late secretory phase (days 24–28) and a significant downregulation of basolateral Scribble during the mid- (days 19–23) and late secretory phase of the menstrual cycle. These findings were taken as an indication of apicobasal polarity loss at a time, when embryo implantation occurs.

Denker, 1990, 1993 [29,30], coined the term “cell biological paradox” to emphasize the highly unusual situation, where two epithelial cells, i.e., the endometrial epithelial cells and embryonic trophoblasts, form junctions through their apical domains [31]. He compared this peculiar state of receptivity with the epithelial–epithelial interactions during embryonal development as they occur, for example, during the closure of the neural tube [32]. Research on neural tube formation revealed that cell and tissue polarity play a major role in neural tube morphogenesis. Apicobasal polarity modulations were described to be regulated by bone morphogenetic proteins (BMPs) and transforming growth factor β (TGFβ) during primary neurulation, where a flat epithelial sheet rolls up into the neural tube [33,34].

The importance of inverted apicobasal polarity was comprehensively summarized in a recent review by Pasquier et al., 2024 [35]. They defined inverted polarity as polarity which occurs when some or all components of the apical and basolateral domains are reversed while the overall polarity axis remains intact. They further stated that inverted polarity is mostly pathological, and that the mammalian embryo implantation represents the only physiological example of this process.

Murphy, 2004 [36], called this phenomenon “plasma membrane transformation” (PMT) and referred to Denker, 1994 [31], who had related this process to epithelial–mesenchymal transition (EMT) [37]. Whitby et al., 2020, highlighted in a recent review the alterations in epithelial cell polarity during endometrial receptivity and compared PMT and EMT [38]. They emphasized similarities in alterations of the actin cytoskeleton, remodeling of cell–cell junctions and cell surface markers (e.g., αVβ3 integrins) but also pointed out major differences in the regulation of tight junctions and in the expression of membrane glycoproteins of the mucin family.

**Figure 3:**
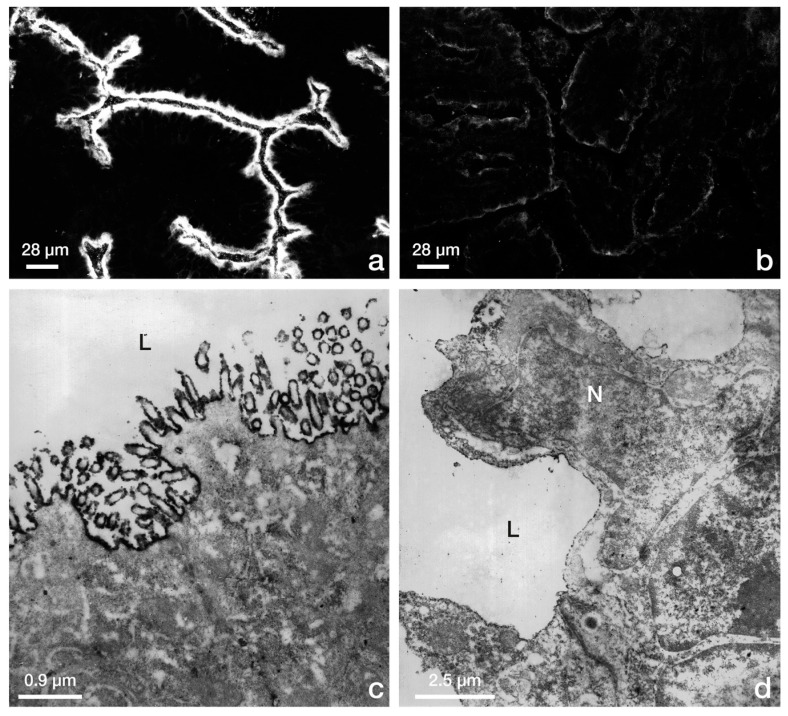
(**a**,**b**) Immunofluorescence staining of the brush border enzyme aminopeptidase M in rabbit luminal endometrial epithelium. (**a**) Pre-receptive state, 3 days post human chorion gonadotropin injection (dp.hCG). Apical plasma membrane is strongly stained. (**b**) Receptive state, 8 dp.hCG. Reactivity is largely lost. (**c**,**d**) Transmission electron microscopy, immunoperoxidase staining of aminopeptidase M in rabbit luminal endometrial epithelium. (**c**) Pre-receptive state, 5 dp.hCG. The apical plasma membrane shows strong reactivity in the microvillar surface epithelium. (**d**) Receptive state, pregnancy, 7 d post coitum (dp.c.). L (Lumen), N (Nucleus). Figure reproduced from [40].

The first hints for the complete remodeling of the endometrial epithelium were obtained in studies of rabbits [30,39,40] and rats [41,42]. During the pre-implantation phase (day 3–5) of pregnant or pseudopregnant rabbits, a strong apical expression of typical brush border enzymes in the luminal endometrial epithelium, i.e., aminopeptidase M (APM or APN) (Figure 3), γ-glutamyl transferase (γ-GT), dipeptidyl peptidase IV (DPPIV) and alkaline phosphatase [39], was detected. Their expression decreased during the implantation phase (day 7–8) in pregnancy or in pseudopregnancy, induced by injection of human chorion gonadotropin (hCG). This has been taken as an indication of a partial loss of polarization.

It has been shown recently that DPPIV and APN are released into the human uterine cavity via shedding of microvesicles from endometrial epithelial cells [43]. Interestingly, extracellular vesicles derived from endometrial cells have been proposed to act on trophectoderm cells to promote implantation [43].

### 2.3 Rearrangement of Adhesion Complexes Occurs in the Endometrial Epithelium During Acquisition of the Receptive State

**Figure 4:**
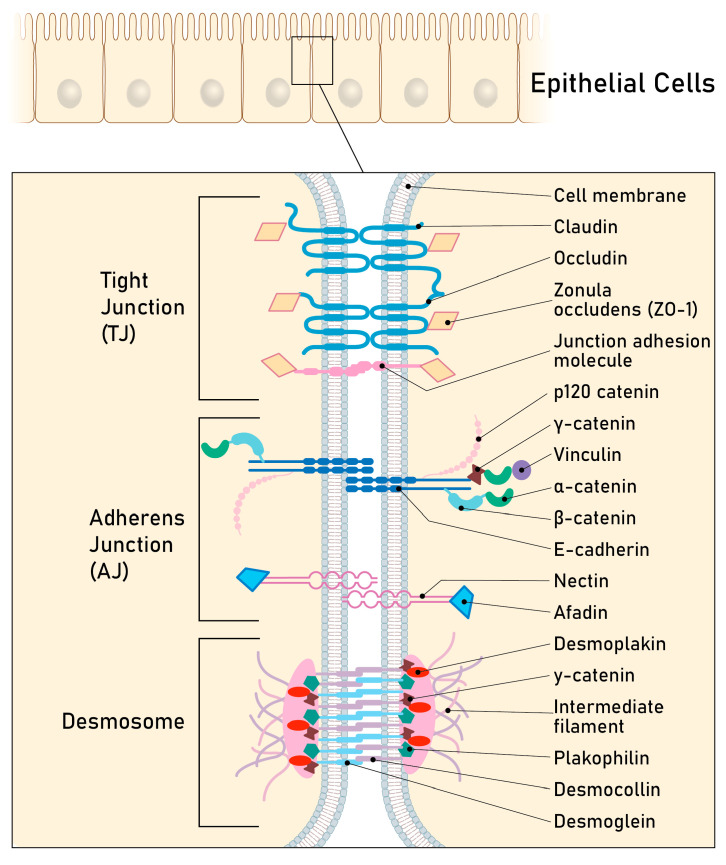
Schematic representation of the cell–cell adhesion complexes in a typical epithelial cell. Tight junctions are at the uppermost part of the lateral cell membrane regulating paracellular transport (gate function) and maintaining cell polarity (fence function). The tight junction complex consists of the transmembrane proteins of the claudin and occludin families and the integral junction adhesion molecules (JAMs) which are associated with cytoplasmic plaque proteins such as zonula occludens, i.e., the protein ZO-1. Immediately basal to the tight junction is the adherens junction. The adhesion is mediated by transmembrane cadherins, mainly E-cadherin [46]. The cadherins bind to cytoplasmic proteins of the catenin family and vinculin, which link the junction to the actin cytoskeleton. Desmosomal complexes [47] are basal to adherens junctions and are involved in strengthening cell–cell adhesion and resisting cellular mechanical stress. They are linked by the desmosomal cadherins of the desmoglein (Dsg) and desmocollin (Dsc) type and are associated with the linker proteins plakophilin and plakoglobin. They are connected to keratin intermediate filaments via the desmosomal plaque protein desmoplakin (Dsp). Scheme modified from [48].

As depicted in Figure 4, epithelial cells are connected by adhesion complexes. The adhesions are formed by a tripartite junctional complex at the subapical circumference of polarized epithelia, first defined and detected by electron microscopy [44]. It consists of the keratin filament-anchoring desmosomes, the actin-associated adherens junctions and the tight junctions, which separate the apical from the basolateral domain [45].

**Figure 5:**
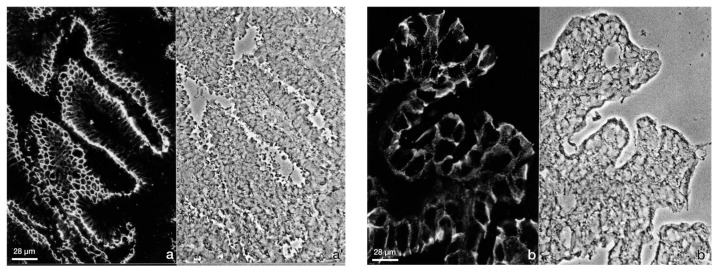
Immunofluorescence staining of the desmosomal plaque protein desmoplakin (Dsp1/2) in rabbit luminal endometrial epithelium. (**a**) Pre-implantation phase (6 dp.c.). Strong subapical staining of the plasma membrane; (**a′**) phase contrast, same region. (**b**) Implantation phase (9 dp.c.). The subapical staining is reduced and Dsp1/2 is localized at the entire basolateral membrane; (**b′**) phase contrast, same region. Figure reproduced from [40].

In addition to the loss of brush border enzymes at the time of embryo implantation in the rabbit, the exclusively subapical staining of the desmosomal plaque protein desmoplakin 1/2 (Dsp 1/2) is reduced in rabbit during the preimplantation phase and the entire basolateral plasma membrane becomes stained during the implantation phase as shown in Figure 5. This redistribution indicates a loss of typical features of apicobasal polarity.

Illingworth et al., 2020 [49], reported comparable results in mice observing a reduction of desmosomes in the luminal uterine epithelium during the preimplantation phase of pregnancy by immunolocalization of Dsp 1/2 and electron microscopy. A loss of desmosomes during early pregnancy was also reported in rat uterine epithelial cells by immunostaining against the desmosomal cadherins desmoglein1/2 (Dsg1/2) and by electron microscopy [50,51].

Schlafke and Enders, 1975 [52], pointed out that modes of implantation are highly species-specific, even among mammals. They distinguished three different trophoblast-endometrial interactions during implantation by transmission electron microscopy.

**The displacement type** of implantation occurs in mice and rats, by which the uterine epithelium is detached from the underlaying basal lamina.

**The fusion type** of implantation takes place in rabbits. Here, the syncytiotrophoblast fuses with the uterine epithelial cells.

**The intrusion type** is most likely the implantation mode of human embryos. In this case, the syncytiotrophoblast penetrates between apparently healthy uterine epithelial cells breaching the intercellular junctions and forming new junctions. Details of this mode have been studied in the ferret [53].

In vitro confrontation studies of primary human endometrial cells with human blastocysts support the notion that the intrusion type of invasion is the main mode of human implantation. Bentin-Ley et al., 2000 [54], could show by using transmission electron microscopy that the trophoblast cells form apical junctional complexes and interdigitations with the apical and lateral plasma membranes of endometrial epithelial cells in vitro. Due to ethical constrictions, only historical tissue slides document human implantation in situ starting from day one after initiation of implantation. They are available in the Carnegie collection [55]. Allen Enders documented the various stages and provided images of this collection. They are accessible through the Loke Centre of Trophoblast Research, University of Cambridge (https://www.trophoblast.cam.ac.uk/Resources/enders, accessed on 20 April 2025). The stages used in that report are from Streeter’s horizons [56,57]. Since the implantation mode between different species differs [52], the studies of epithelial surface epithelium in mice, rats or rabbits cannot be completely transferred to the human situation. Nevertheless, certain aspects of the transformation of the apical and lateral plasma membranes are comparable (reviewed in [58]).

**Figure 6:**
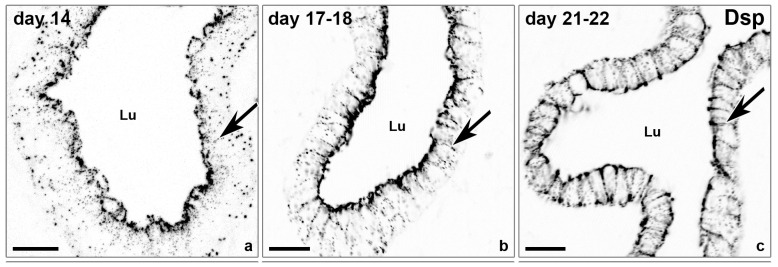
Confocal fluorescence micrographs (inverse presentation; single focal planes). Immunofluorescence staining of the desmosomal plaque protein Dsp1/2 on glandular endometrial epithelial cells at different times of the menstrual cycle (**a**–**c**); arrows pointing to single desmosomes. Lu (Lumen). Scale bar: 20 µm. The images were modified from [59].

To gain more insight into the changes of adhesion proteins in human endometrial epithelial cells, the expression of components of desmosomes, adherens junctions and tight junctions have been examined by our group during different stages of the menstrual cycle [59]. A redistribution of the desmosomal plaque protein desmoplakin 1/2 (Dsp1/2) (Figure 6) and the desmosomal cadherin desmoglein 2 (Dsg2) to the entire basolateral membrane were found at the time of implantation [59].

In the proliferative phase (day 14) and the early secretory phase (day 17–18), a subapical enrichment of Dsp1/2 could be detected. At day 21/22, i.e., at the time of implantation, the exclusively subapical staining was reduced and the entire basolateral plasma membrane was stained for Dsp1/2. This is in accordance with investigations of the human endometrial apical glycoproteomic [60]. Dsg2 was located on the apical, lateral and basal side of luminal epithelial cells in the mid-secretory phase endometrium. Sarani et al.,1999 [18], asserted that the proportion of cell membrane occupied by desmosomes was significantly reduced 6 days after the luteinizing hormone surge (LH+6) in the implantation window.

**Figure 7:**
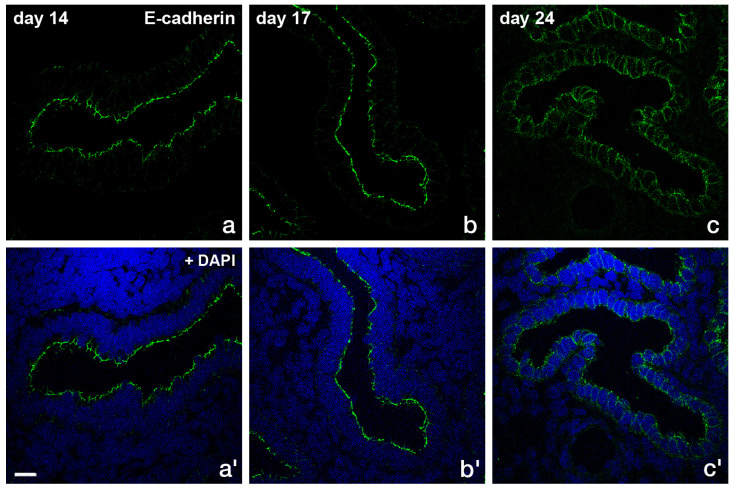
Immunofluorescence staining of the adherens junction protein E-cadherin on glandular epithelial cells at different times of the menstrual cycle (**a**–**c**; **a′**–**c′** with DAPI staining). Scale bar: 20 µm. The images were modified from [59].

**Figure 8:**
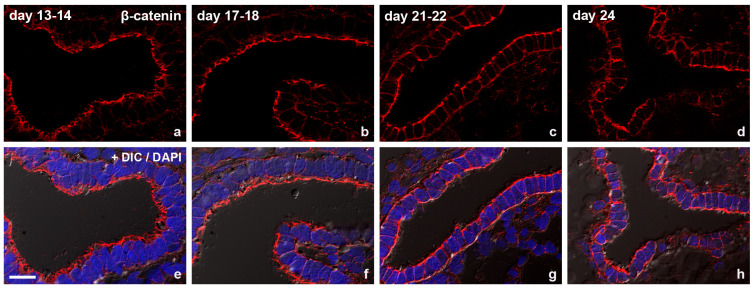
Immunofluorescence staining of the adherens junction protein β-catenin on glandular epithelial cells at different times of the menstrual cycle (**a**–**d**; with DAPI staining in **e**–**h**). Scale bar: 20 µm. The images were modified from [59].

The adherens junction transmembrane protein E-cadherin (Figure 7) and the intracellular anchor protein β-catenin (Figure 8) were also shown to be redistributed from the typical subapical position to the basolateral plasma membrane during the implantation phase [59].

A strong subapical staining was obvious in the proliferative phase (day 14) and early secretory phase (day 17). In the secretory implantation phase, E-cadherin was redistributed along the lateral plasma membrane.

For β-catenin, a strong subapical staining in the proliferative phase (day 13–14) and early secretory phase (day 17–18) was detected. In the secretory implantation phase (days 21/22 and day 24), β-catenin was redistributed along the lateral plasma membrane.

The tight junction component ZO-1 was localized by immunofluorescence at the uppermost part of the lateral membrane and did not change in distribution or staining intensity during the menstrual cycle [59]. Using freeze-fracture electron microscopy, changes in tight junctions during the menstrual cycle were shown by Murphy et al. [61,62]. A reduced extension of the tight junctions was detected in the secretory phase (0.21 µm vs. 0.43 µm in the proliferative phase).

As reviewed by Grund and Grümmer, 2018 [63], the role of claudins (Cldns) is important for implantation. The claudin content determines the permeability characteristics. Thus, the combination and ratio of the different claudins may be an important factor controlling embryo implantation. Studies in Cldn 3 knockout mice revealed a reduced number of implantation sites, accompanied by a reduced depth of ectoplacental cone invasion [64].

During the menstrual cycle, Cldn 3 and Cldn 10 were immunohistochemically localized in the apical part of the lateral membrane in glandular and luminal epithelium. No obvious differences in staining intensity and localization could be detected between the proliferative and secretory phase [65]. This might be due to the low resolution of light microscopy in contrast to the results obtained by freeze-fracture electron microscopy. Interestingly, Zhang et al., 2024, reported in a recent study [66] that the expression of the tight junction proteins Cldn 3 and Cldn 4 was dysregulated in patients with hyperlipidemia, a condition which is associated with impaired fertility. qRT-PCR results showed upregulation of Cldn 3 and downregulation of Cldn 4 expression.

Elevated testosterone levels in women with the endocrine disorder polycystic ovary syndrome (PCOS) influence the integrity of tight junctions in the endometrial epithelium by decreasing Cldn4 and occludin [67]. Similarly, administration of testosterone in ovariectomized rats caused loss of tight junction complexity and downregulated expression of Cldn4 and occludin in the uterus [68]. This was shown to have significant implications for the process of embryo attachment and subsequent implantation in rats [69].

## 3. Part II: In Vitro Observations

### 3.1. The Degree of Polarization and Adhesion Differs Between Human Endometrial Epithelial Cell Lines

#### 3.1.1. Studies with Monolayers

Since studies of early human implantation are restricted because of ethical constraints, suitable in vitro models are crucial and have been established by using primary endometrial cells and endometrial epithelial cell lines. To account for the different polarized stages during the menstrual cycle, endometrial cell lines were chosen with different degrees of polarization [70,71,72], review in [73], notably,

(1) Highly polarized HEC-1-A cell line, established by [74];
(2) Moderately polarized Ishikawa cell line, established by [75,76];
(3) Poorly polarized RL95-2 cell line, established by [77].

Studying these cells as confluent monolayers, John et al., 1993 [72], and Thie et al., 1995 [70], first characterized the epithelial phenotype of HEC-1-A and RL95-2 cells and tested for adhesive or non-adhesive behavior of the apical pole towards human JAR choriocarcinoma cells. HEC-1-A cells were identified as non-adhesive and RL95-2 cells as adhesive. The cell lines were then examined by electron microscopy and immunohistochemistry [78,79] and were used as in vitro models for receptive (RL95-2) and non-receptive uterine epithelial cells (HEC-1-A) [71]. It was postulated that the loss of epithelial cell polarity is a prerequisite for adhesiveness.

Atomic force microscopy was used to further study the adhesion of HEC-1-A and RL95-2 with JAR cells [80,81]. Measuring the interaction forces between the cantilever-attached JAR trophoblast cells and the uterine epithelial monolayers only, the poorly polarized cell line RL95-2 attached strongly to trophoblast-type cells via their apical cell poles. Thie et al. suggested that the lack of tight junctions and the rudimentary adherens junctions, together with the non-polar organization of the actin cytoskeleton, accounted for this effect and explained the differences with the more polarized HEC-1-A cells.

According to Heneweer et al. [82,83], the small GTPase RhoA-mediated reorganization of the actin cytoskeleton was crucial for binding of JAR cells to the RL95-2 cell line. Trophoblast binding led to a marked increase in F-actin and to an increase in colocalized RhoA. Inhibition of Rho-dependent signaling in RL95-2 cells by treatment with Clostridium difficile toxin A impeded binding of trophoblastic cells. In contrast to the results obtained with RL95-2 cells, the moderately polarized Ishikawa cells remained adhesive for JAR spheroids [84].

**Figure 9:**
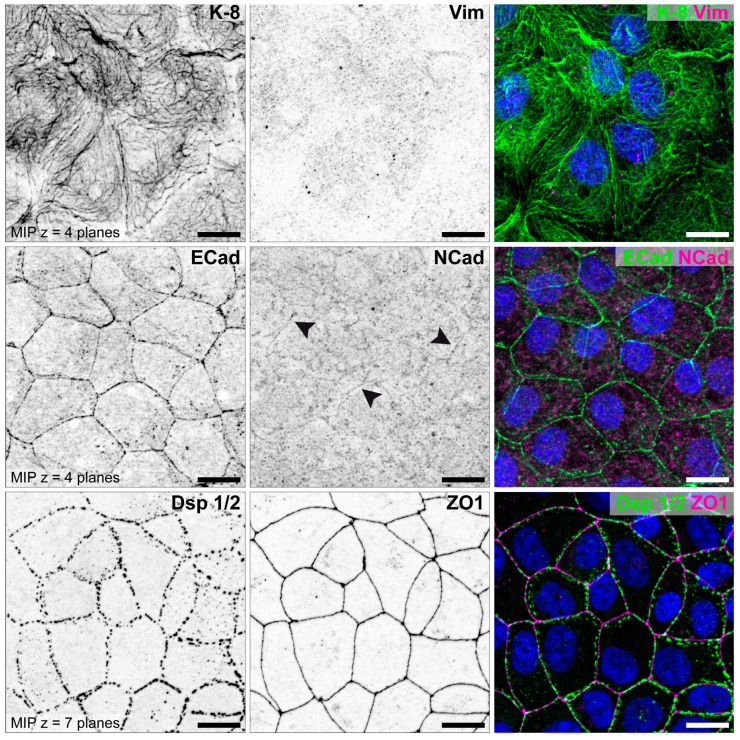
Characterization of endometrial epithelial cell line HEC-1-A. Cells were grown for 5 days on glass and double stained for intermediate filaments keratin 8 (K-8, green) and vimentin (Vim, magenta), adherens junction proteins E-cadherin (ECad, green) and N-cadherin (NCad, magenta) (arrowheads), desmosomal protein desmoplakin (Dsp 1/2, green) and tight junction protein ZO1 (magenta). Nuclei were counterstained with Hoechst 33342 (blue). Single fluorescent channels are displayed as inverse representation. Cells are positively stained for epithelial-specific keratin intermediate filament polypeptides but not for the mesenchymal intermediate filament polypeptide vimentin. At lateral membranes, HEC-1-A cells show characteristic staining patterns for the three epithelial cell junctions ECad, Dsp 1/2 and ZO1. Scale bar = 25 μm. Structured illumination microscopy for optical sectioning was performed using a Zeiss ApoTome.2 mounted to an Axio Imager M.2 microscope, Carl Zeiss, Jena, Germany. Figure reproduced from [85].

**Figure 10:**
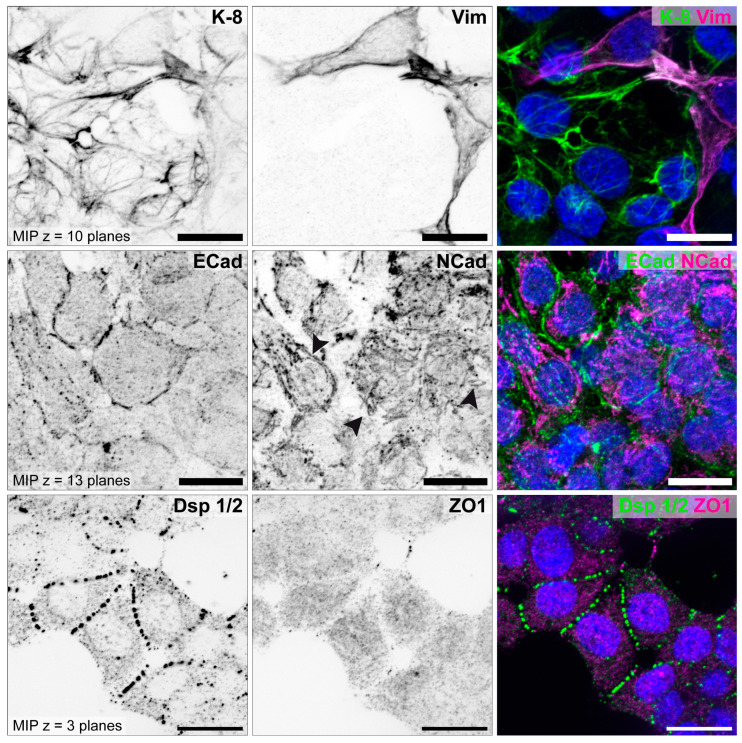
Characterization of endometrial epithelial cell line RL95-2. Cells were grown for 5 days on glass and double stained for intermediate filament polypeptides keratin 8 (K-8, green) and vimentin (Vim, magenta), adherens junction proteins E-cadherin (ECad, green) and N-cadherin (NCad, magenta), and desmosomal protein desmoplakin (Dsp 1/2, green) and tight junction protein ZO1 (magenta). Nuclei were counterstained with Hoechst 33342 (blue). Single fluorescence channels are displayed as inverse representation. Cells are positively stained for K-8. In addition, some cells exhibit a vimentin network, have a continuous ECad signal and are stained positive for NCad (arrowheads). RL95-2 cells show a punctate signal of Dsp1/2 but no specific signal for ZO1. Scale bar = 15 μm. Structured illumination microscopy for optical sectioning was performed using a Zeiss ApoTome.2 mounted to an Axio Imager M.2 microscope. Figure reproduced from [85].

**Figure 11:**
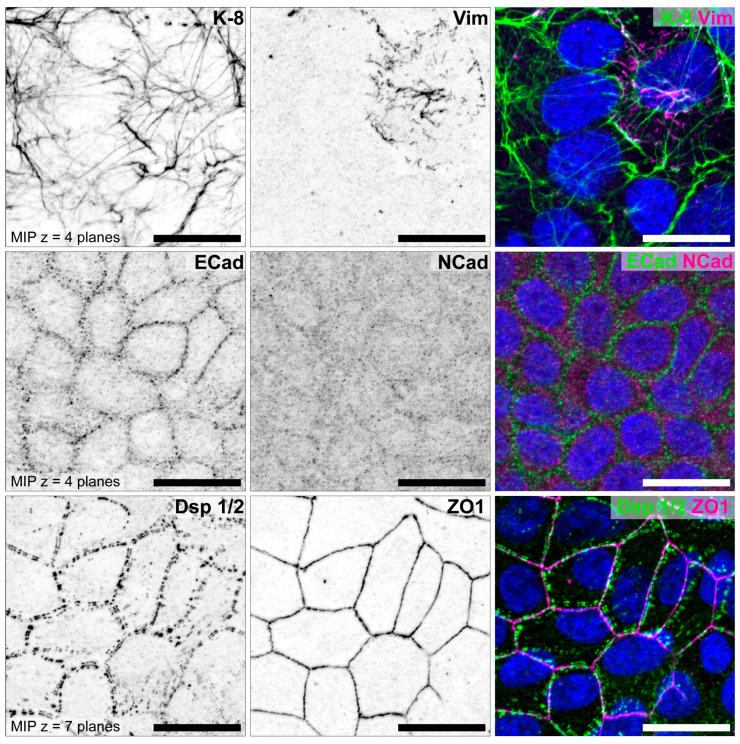
Characterization of endometrial epithelial cell line Ishikawa. Cells were grown for 5 days on glass and double stained for intermediate filament polypeptides keratin 8 (K-8, green) and vimentin (Vim, magenta), adherens junction proteins E-cadherin (ECad, green) and N-cadherin (NCad, magenta), and desmosomal protein desmoplakin (Dsp 1/2, green) and tight junction protein ZO1 (magenta). Nuclei were counterstained with Hoechst 33342 (blue). Single fluorescence channels are displayed as inverse representation. Scale bar = 10 μm. Structured illumination microscopy for optical sectioning was performed using a Zeiss ApoTome.2 mounted to an Axio Imager M.2 microscope. Figure reproduced from [85].

In addition to the studies of Thie et al. and Heneweer et al., the cell lines were further characterized by our group for N-cadherin [85]. Representative illustrations for all three cell lines are shown in Figure 9 (HEC-1-A), Figure 10 (RL95-2) and Figure 11 (Ishikawa) [85].

The staining of the two cell lines differs clearly. The strongly polarized HEC-1-A endometrial cell line show distinct immunoreactions for components of the tripartite junctions, but no staining for vimentin, which is produced in cells of mesenchymal origin, and a very weak staining for N-cadherin. In contrast, the poorly polarized RL95-2 cell line presents a reduced keratin network and an increased vimentin network. E-cadherin is weakly expressed and N-cadherin clearly visible. Desmosomes are reduced and show a punctate Dsp1/2 signal. Tight junctions cannot be detected using ZO1 antibodies.

The well differentiated but moderately polarized Ishikawa cell line shows positive staining for keratin 8. Single cells within the monolayer show an additional vimentin network. ECad, Dsp 1/2 and ZO1 are localized at lateral cell membranes and a very weak staining for NCad is visible.

**Figure 12:**
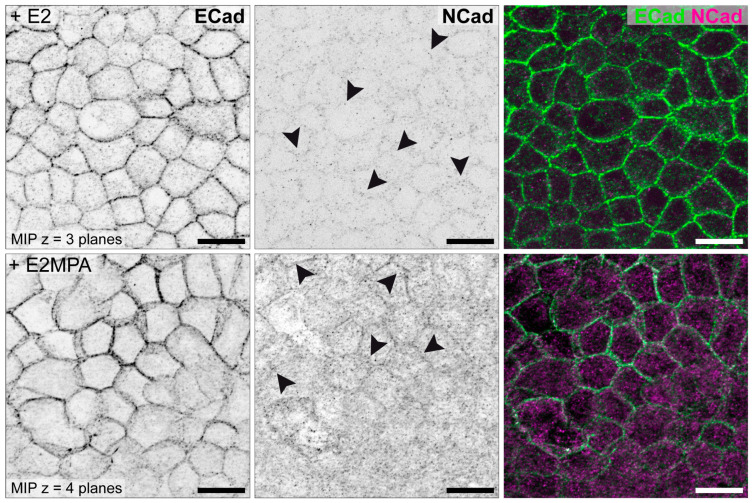
Characterization of endometrial epithelial cell line Ishikawa under hormonal stimulation. Representative immunofluorescence staining of E-cadherin (ECad, green) and N-cadherin (NCad, magenta) in Ishikawa cells after 5 days of stimulation with either 17β-estradiol (E2) or the combination of E2 and medroxyprogesterone acetate (MPA). In E2-treated cells, NCad shows a weak punctate signal (arrowheads), whereas it shows a continuous location at the cell borders in cells treated with E2/MPA (arrowheads). Single fluorescence channels are displayed as inverse representation. Scale bar = 15 µm. Structured illumination microscopy for optical sectioning was performed using a Zeiss ApoTome.2 mounted to an Axio Imager M.2 microscope. Figure reproduced from [85].

Hormonal stimulation of the Ishikawa cells in monolayer culture as shown in Figure 12 was performed to mimic the proliferative and secretory phase of the menstrual cycle. E2 treatment does not change the weak punctate NCad signal, whereas E2/MPA treatment mimicking the secretory phase increases the N-cadherin immunofluorescence, which becomes localized in continuous lines at cell–cell borders (Figure 12). This indicates that E2/MPA treatment induces a switch to a more mesenchymal cell identity in Ishikawa cells.

To look for the integrity of interepithelial tight junctions as an indicator of cellular polarity, Whitby et al., 2018 [28], measured the transepithelial resistance (TER) of Ishikawa monolayers after treating them with estrogen and progestin. They found that the resistance increased with estrogen supplementation and decreased after treatment with additional progestin, simulating the different hormonal situations during the menstrual cycle.

The reduction in extracellular matrix stiffness during decidualization [86] is believed to influence the mechanical properties of the endometrial epithelium [23]. To study this effect in vitro, human endometrial Ishikawa cell monolayers were grown on substrates with different stiffness by our group [87]. Using hydrogels of defined stiffness, effects on cell morphology were observed. Thus, lower substrate stiffness resulted in increased cell height and increased number of cells per area.

A summary of the different expressions of keratin 8, vimentin, Ecad, Ncad, Dsp1/2 and ZO1 in HEC-1-A, RL95-2 and Ishikawa cells is listed in Table 1 together with the corresponding references.

#### 3.1.2. Studies with 3D Endometrial Epithelial Spheroids

**Figure 13:**
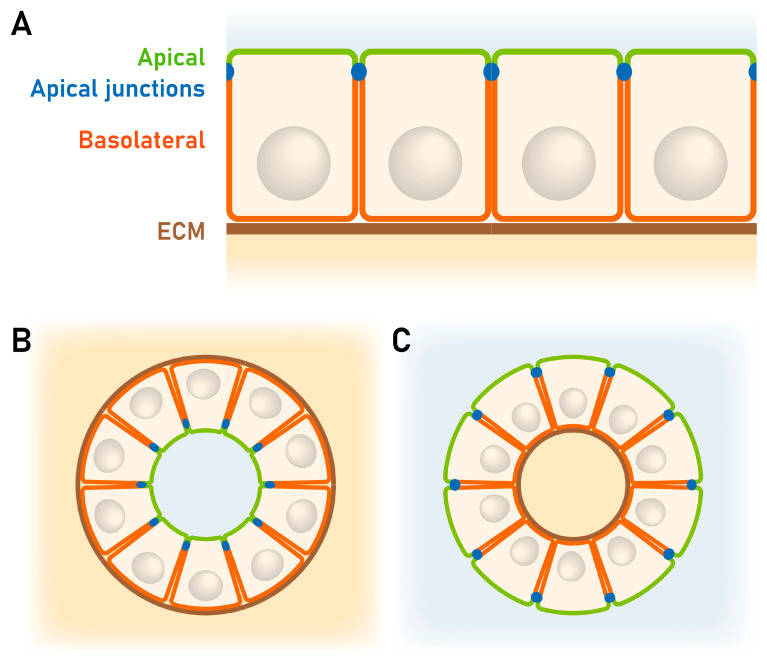
Apical–basal properties of epithelial cells in 2D or 3D culture. (**A**) Polarized epithelial cell monolayer on extracellular matrix. Apical junctions separate the basolateral membranes from the apical membrane. (**B**) Apical-in spheroids. Polarized epithelial cells in an ECM gel forming a spheroid with the apical side in and the basal side on the outside surface facing the ECM. (**C**) Apical-out spheroids. Polarized epithelial cells forming a spheroid in suspension or in agarose molds. Apical membranes are formed on the outside surface of the spheroid and the basolateral membranes are formed inside. Scheme modified from [90].

Effects of different cell polarization can be better observed in 3D than in 2D culture systems [90]. Moreover, the 3D culture in an extracellular matrix (ECM) gel is closer to physiological conditions. Therefore, experiments have been performed with differently polarized cell lines growing in reconstituted basement membrane (Matrigel) as depicted in the schematic drawing (Figure 13B).

There are different ways to culture 3D spheroids with apical–basal polarity [90]. When epithelial cells are cultured in ECM gels as Matrigel, they form spheroids with basal membranes towards the ECM and apical membranes at the inner surface typically forming a central lumen (Figure 13B). Hence, in thick Matrigel droplets, endometrial spheroids can be grown with apical-inside polarity as a model for glandular structures [85,89,91]. In contrast, when epithelial cells are cultured in suspension (hanging drop culture) in a shaking system or in agarose molds, they exhibit apical membranes at the outer surface and produce their own ECM, which is in the inside of the spheroid (Figure 13C). Trophoblast-derived AC-1M88 spheroids have been generated in this way as a tool for studying adhesion to the Ishikawa cell monolayer [87] and to study transepithelial trophoblast invasion in Ishikawa monolayer cultures. Earlier studies have used this method to examine the first contact between trophoblast and the different polarized endometrial cell lines by using apical-out spheroids of choriocarcinoma cell lines [92]. Grümmer et al., 1994 [93], generated spheroids of three human choriocarcinoma cell lines (BeWo, Jeg-3 and Jar) and performed adhesion studies with human endometrial explants of the mid-secretory phase instead of cell lines. Interestingly, these three choriocarcinoma cell lines differed in adhesion to uterine epithelium and in invasion to endometrial stroma, although they were all invasive in a general invasion assay. 

**Figure 14:**
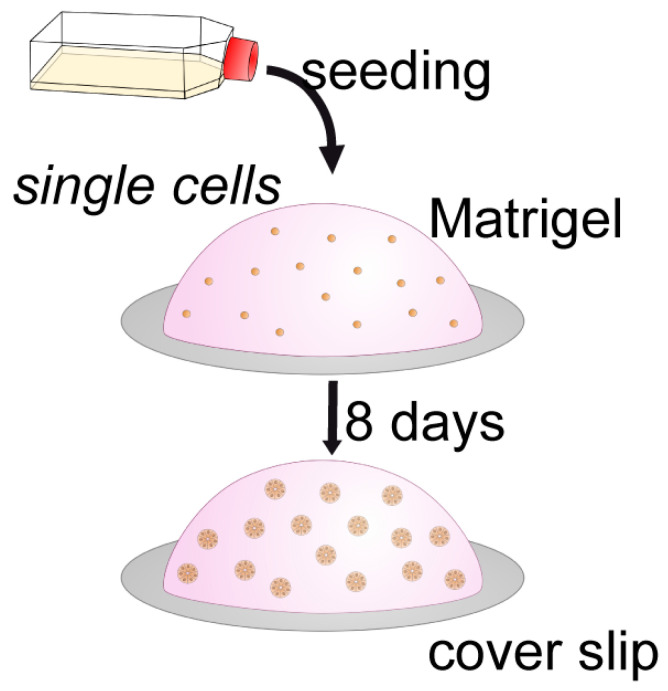
Scheme of the culture system for apical-in spheroids. Single endometrial cells are resuspended in serum-free, Phenol Red-free DMEM/F-12 medium and carefully mixed with (growth factor reduced) GFR-Matrigel^®®^ in a ratio of 1:1. Cell–gel droplets of 25 μL are plated on glass coverslips and allowed to polymerize in the incubator for 20 min. Cells are then incubated in normal growth medium for 8 days. In the gel, the cells formed multicellular, single-layered spheroids. Spheroids displayed classical features of apical-in epithelial polarity as detected by ZO1 and Dsp1. Figure reproduced from [85].

**Figure 15:**
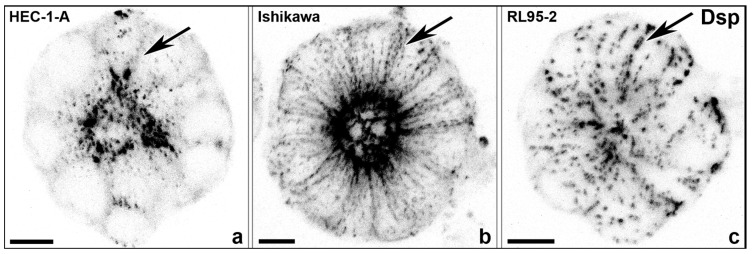
Gland-like, apical-in spheroids were obtained in Matrigel droplets. (**a**) Highly polarized cell line HEC-1-A, (**b**) intermediate polarized Ishikawa cell line, (**c**) low-polarized cell line RL95-2. Confocal fluorescence micrographs (inverse presentation; projections of 8 consecutive focal planes) reveal desmosomal anti-desmoplakin (Dsp) reactivity. The entire basolateral staining of desmosomal cell–cell adhesion (see arrows) occurs in the less polarized epithelial cell lines Ishikawa and most notably in RL95-2 comparable to the decrease in polarization during the menstrual cycle, where at day 21/22 at the time of implantation, the exclusively subapical staining is reduced, and the entire basolateral plasma membrane is stained for Dsp1/2. Scale bars: 10 µm. The images were modified from [23,89].

**Figure 16:**
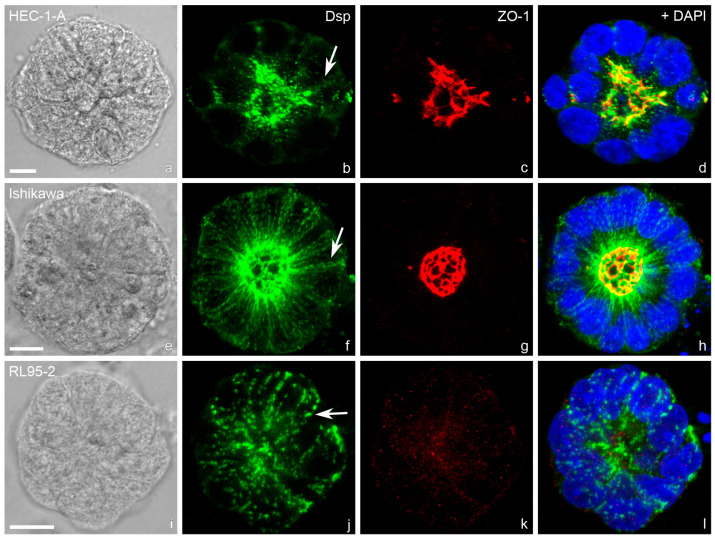
Spheroids of the three different endometrial epithelial cell lines after 9 days in Matrigel are shown. Immunofluorescence staining was performed to detect desmoplakin 1/2 (Dsp) and ZO-1. Z-projections of eight consecutive confocal planes through the middle of typical spheroids are presented. Differential interference contrast micrographs are shown in (**a**,**e**,**i**). Fluorescence pictures were merged and combined with nuclear staining (DAPI, blue) in (**d**,**h**,**l**). Arrows in (**b**,**f**,**j**) point to lateral plasma membranes of adjacent epithelial cells. In **HEC-1-A** spheroids the dot-like staining of the desmosomal plaque protein Dsp (green) is concentrated in the subapical regions of lateral plasma membranes. Towards the basal margins of lateral membranes, the desmosomal dots are strongly decreased in number (**b**). **Ishikawa** spheroids also showed a subapical concentration of desmosomes. The density of desmosomal dots is highest in the subapical region and sparser along the lateral plasma membrane. In contrast to HEC-1-A spheroids, however, there is no decrease in desmosomes towards the basal margins (**f**). Both HEC-1-A and Ishikawa spheroids form lumina surrounded by the tight junctional protein ZO-1 (red stain; **c**,**g**). In contrast, lumen formation and the tight junction protein ZO-1 are not detectable in **RL95-2** spheroids (**k**). The desmosomes of RL95-2 spheroid cells are evenly distributed along the entire lateral plasma membranes and showed no concentration towards the center of the spheroids (**j**). The distribution of desmosomes correlates with the proposed polarity of human endometrial epithelial cell lines. Scale bars represent 10 µm and apply to all images in each row. Figure reproduced from [89].

A scheme of the culture system for generating apical-in spheroids is shown in Figure 14 and gland-like, apical-in spheroids obtained in Matrigel droplets are presented in Figure 15 and Figure 16.

### 3.2. Hormone Responses Can Be Studied in 3D Endometrial Epithelial Spheroids

**Figure 17:**
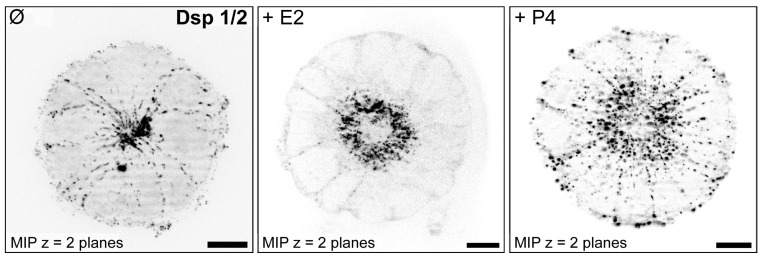
Immunofluorescence staining of desmoplakin 1/2 (Dsp), inverse representation projection views. Beginning from day 4 of incubation, Ishikawa spheroids were stimulated with estrogen (E2) or progesterone (P4). Spheroids were stained after 8 days of incubation. Structured illumination microscopy for optical sectioning was performed using a Zeiss ApoTome.2 mounted to an Axio Imager M.2 microscope. Images were displayed as MIP = maximum intensity projection. Scale bar = 10 μm. Figure adapted from [85].

**Figure 18:**
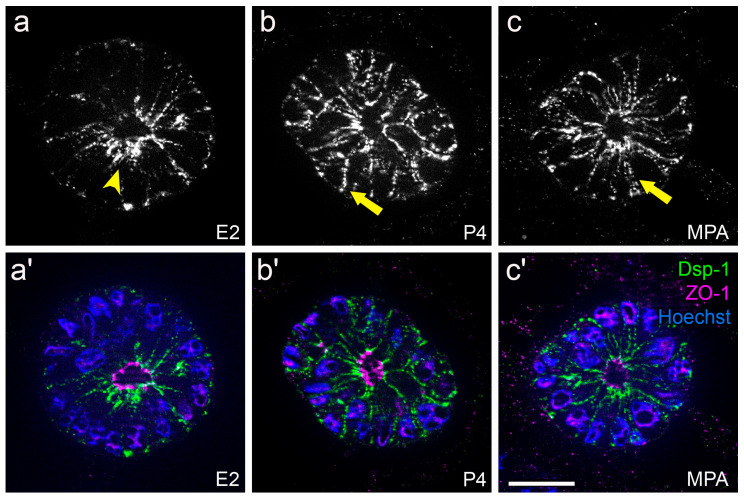
Influence of ovarian steroid hormones on localization of desmoplakin 1 (Dsp-1). Images show Ishikawa spheroids after 4 days of stimulation with estradiol/E2 (**a**,**a′**), progesterone/P4 (**b**,**b′**) or medroxyprogesterone acetate/MPA (**c**,**c′**). Arrowhead highlights subapical accumulation of Dsp-1 expression (**a**). Arrows highlight Dsp-1 redistribution to the basolateral membrane (**b**,**c**). Greyscale pictures for Dsp-1 (**a**–**c**) or in green combined with tight junctional staining (ZO-1, magenta) and Hoechst (blue) in (**a′**–**c′**). Scale bar: 20 μm. Figure adapted from [91].

**Figure 19:**
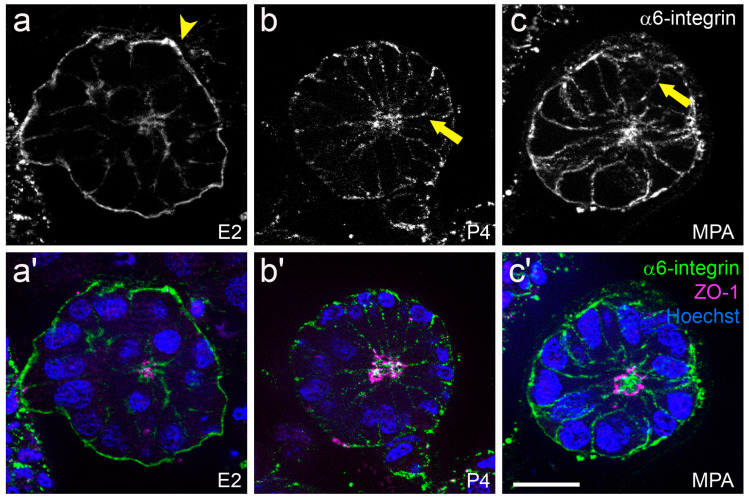
Influence of ovarian steroid hormones on localization of α6-integrin. Images show Ishikawa spheroids after 2 days of stimulation with E2/estradiol (**a**,**a′**), P4/progesterone (**b**,**b′**) or medroxyprogesterone acetate/MPA (**c**,**c′**). Arrowheads highlight the basal localization of α6-integrin in (**a**). Arrows highlight the lateralization of the α6-integrin signal in (**b**,**c**). Greyscale pictures for α6-integrin (**a**–**c**) or in green combined with tight junctional staining (ZO-1, magenta) and Hoechst (blue) in (**a′**–**c′**). Scale bar: 20 μm. Figure adapted from [91].

For studying hormonal regulation of endometrial epithelial polarity in vitro, the Ishikawa cell line was selected as it represents an intermediate state of epithelial polarity and expresses estrogen receptors (ERs) and progesterone receptors (PRs) [85,88,91]. It was proposed that hormonal stimulation induces a switch from a polarized to a less polarized state in these cells. In accordance, Figure 17 and Figure 18 illustrate that Ishikawa spheroids treated with estrogen (E2) or progestin (P4 or MPA) showed a redistribution of the desmosomal plaque protein desmoplakin by progestin treatment to the entire basolateral membrane as it has been demonstrated for the mid-secretory phase of the menstrual cycle in vivo and in the constitutively low-polarized cell line RL95-2 [89]. In addition, the extracellular matrix adhesion receptor α6-integrin was also redistributed from the basal plasma membrane to the entire basolateral membrane in response to progestin treatment (Figure 19). This is in accordance with the changes occurring during the mid-secretory phase of the menstrual cycle described by Albers et al., 1995, and Buck et al., 2021 [91,94].

A limiting factor for this model is the fact that only about 70% of the spheroids behave in this way [91]. The reason is probably that not all spheroids which are derived from single cells are equipped with a functional progesterone receptor [85]. In the future, it would therefore be useful to measure progesterone receptor expression in the spheroids, e.g., by immunostaining. This would help to understand differences in spheroid behavior.

### 3.3. Endometrial Receptivity Can Be Examined in Confrontation Assays with Trophoblasts

#### 3.3.1. Confrontation of Endometrial Epithelial Spheroids with Trophoblast Cells from the Basal Cell Pole

**Figure 20:**
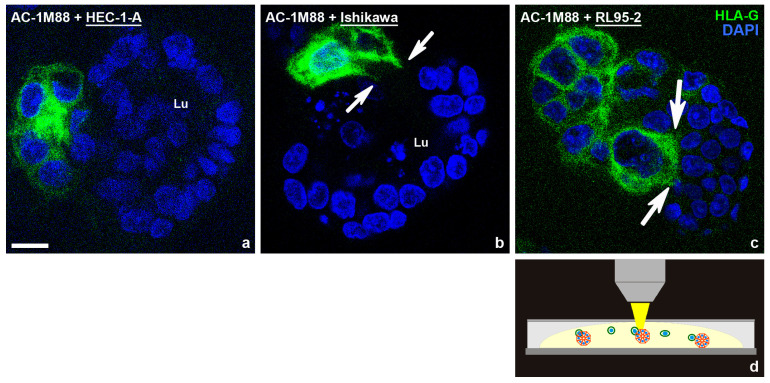
Three-dimensional invasion assay: Human trophoblast–endometrial interaction of trophoblast-derived AC-1M88 cells specifically stained with the human histocompatibility antigen (HLA-G, green) with spheroids of different polarity from the basal cell pole side after 9 days in Matrigel. DAPI (blue) for nuclear staining. (**a**) No invasion of attached AC-1M88 cells into the highly polarized HEC-1-A spheroids occurs. (**b**) AC-1M88 cells partially intercalate between Ishikawa cell spheroids, extending cellular processes into the spheroid. Spheroid lumen (Lu). (**c**) AC-1M88 trophoblast cells completely invade poorly polarized RL95-2 spheroids. Arrows in (**b**,**c**) point to extensions of the trophoblast cells. Scale bar: 10 µm. (**d**) AC-1M88 trophoblast cells were added on day 4 after spheroids had formed. The images were taken after 5 additional days in Matrigel. Figure modified from [89].

To prove the concept that differently polarized spheroids are more or less permissive for invasion of trophoblast cells, an invasion assay was established. As a model for extravillous trophoblast cells invading endometrial glands from the basal side, the AC-1M88 trophoblast cell line was used, which is a hybridoma of primary extravillous trophoblast cells and the JEG-3 choriocarcinoma cell line [95]; review in [73]. They were confronted with the differently polarized endometrial cell-line derived spheroids. As depicted in Figure 20, trophoblast cells were found to be barely attached to the highly polarized HEC-1-A cells, extended cell protrusions into Ishikawa spheroids and completely invaded RL95-2 spheroids.

#### 3.3.2 Confrontation of Trophoblast Spheroids with the Apical Cell Pole of Ishikawa Cell Monolayers

**Figure 21:**
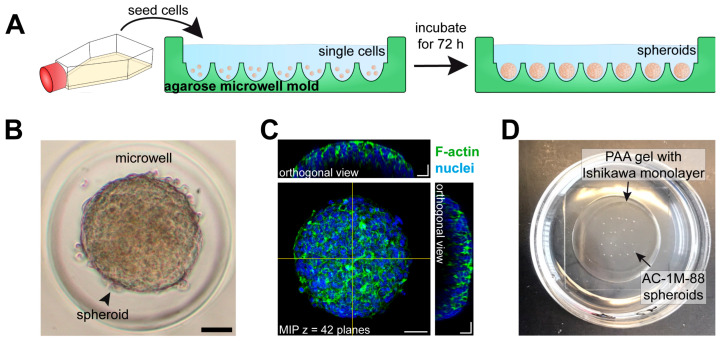
(**A**) The scheme depicts how trophoblast apical-out spheroids are prepared in scaffold-free agarose microwell molds. Agarose molds are custom-made by pouring 3.5% agarose onto a green-colored silicone mold. After sterilization by UV light, AC-1M88 cells (~780 cells/microwell) were seeded into the agarose microwell molds, where they aggregated and formed multicellular spheroids after 72 h of incubation. (**B**) Representative bright field image of an AC-1M88 trophoblast spheroid in an agarose microwell. Scale bar = 50 μm. (**C**) Fluorescence image showing phalloidin-labeled F-actin (green) and Hoechst 33342-stained nuclei (blue) in a trophoblast spheroid. Corresponding orthogonal projections show the cross-sections of the spheroid, as indicated by the yellow lines. Images are displayed as maximum intensity projection (MIP). Scale bars = 50 μm (MIP) and 25 μm (orthogonal views). (**D**) The photograph shows trophoblast spheroids that had been placed manually on top of an Ishikawa monolayer at regular intervals using an embryo transfer pipettor without moving the dish to avoid displacement of the spheroids. After 2 h of incubation, the cells were washed 3× with PBS a xd the remaining adhesive spheroids were counted. PAA (polyacrylamide). Figure reproduced from [87].

To enable the 3D visualization of the adhesion and invasion process, Sternberg and Izmaylova et al. [87] generated apical-out spheroids from the AC-1M88 trophoblast cell line to study their attachment to and invasion of a monolayer of human Ishikawa cells. The procedure of preparing apical-out endometrial spheroids is shown in Figure 21.

**Figure 22:**
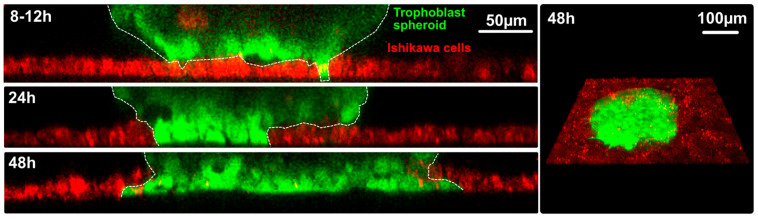
Trophoblast spheroid invasion site. AC1-M88 trophoblast cells were pre-stained with CellTracker Green (Thermo Fisher, North Ryde, NSW, USA), Ishikawa Scientific Inc., Waltham, MA, USA cells with CellTracker Deep Red (Thermo Fisher). Ishikawa Scientific Inc., Waltham, MA, USA cells are growing on Matrigel-coated polyacrylamide gels with a stiffness of 4 kPa. During transepithelial invasion, trophoblast cells displace Ishikawa cells. Z-projection of invasion during different time points on the left. Invasion at 48 h is visualized from the basal side on the right. Figure kindly provided by Liubov Izmaylova, 2025, unpublished.

In a first approach, the adhesion of the trophoblast spheroids on Ishikawa monolayers which were grown on substrates of different stiffness was studied. Higher substrate stiffness increased trophoblast adhesion, emphasizing the role of mechanical cues provided by extracellular matrix [87]. Further ongoing studies focus on the transepithelial invasion and intercellular adhesion between trophoblast spheroids and human endometrial Ishikawa cells. To this end, Ishikawa cells are grown on soft polyacrylamide hydrogels (4 kPa) according to the approximate stiffness of endometrium in vivo [96]. To distinguish the AC-1M88 spheroid-derived invading cells from the endometrial monolayer, each cell type was pre-stained with different live cell dyes prior to confrontation (Figure 22).

Transepithelial invasion is a prerequisite for successful implantation and yet remains one of the less studied events. Besides the study of Bentin-Ley et al., 2000 [54], most studies focused mainly on adhesion of the blastocyst to the endometrial epithelial cells, while the penetration of the trophoblast through the epithelial cell layer is not well documented [97] and is not sufficiently understood at the molecular and mechanistic levels. Only one previous study, by John et al., 1993 [72], revealed transepithelial invasion of JAR spheroids into the poorly polarized RL95-2 epithelial cell monolayer and detected membrane contacts between JAR and RL95-2 cells by electron microscopy.

Also, a recent paper [98] focused on transepithelial invasion. Using human blastocysts, the authors could show that trophectoderm-derived syncytiotrophoblasts penetrate the Ishikawa cell layer. Through interactions with endometrial epithelial cells, the trophoblast cells differentiated into multinucleated syncytiotrophoblast cells.

## 4. Discussion

**Figure 23:**
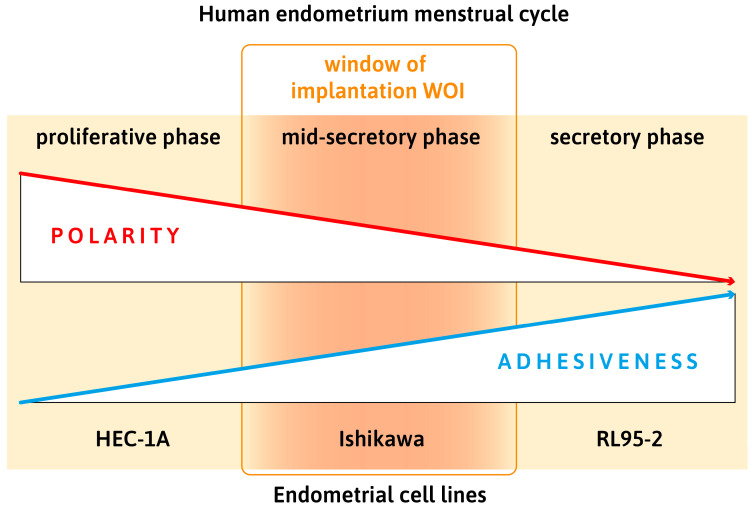
Simplified summary scheme highlighting key properties of three endometrial cell lines representing different stages of the menstrual cycle. HEC-1A cells have highest polarity, Ishikawa moderate, and RL95-2 lowest polarity with inverse behavior for adhesiveness. The epithelial cells of human endometrium during the menstrual cycle exhibit highest polarity during the proliferative phase and decreasing polarity during the secretory phase and conversely increasing adhesiveness and receptivity during the mid-secretory phase, i.e., the window of implantation. The Ishikawa cells with functional estrogen and progesterone receptors can be switched in cell culture by progesterone treatment to a less polarized state. This is evidenced by the redistribution of desmosomal proteins to the entire basolateral membrane, which reflects the changes observed during the mid-secretory phase of the menstrual cycle and the distribution in the constitutively low polarized cell line RL95-2.

To become receptive for an embryo, the endometrium undergoes substantial remodeling during each menstrual cycle [58]. The concepts of “loss of polarity” and “plasma membrane transformation” (PMT) as prerequisites for successful implantation, proposed by Denker [29,30,31,70,78] and Murphy [36,41,42], have stimulated abundant research and gained wide acceptance [21,28,38,58,59,82,83,89,91,99,100,101]. We focused in this review on changes in adhesion, summarizing our own results and the recent literature. The redistribution of desmosomal and adherens junctions in human endometrial epithelial cells during the progesterone-dominated secretory phase of the menstrual cycle has been demonstrated [59] and the benefit for using different polarized endometrial epithelial cell lines in a monolayer or in a 3D culture system has been presented. Figure 23 summarizes schematically how they relate to different phases of the menstrual cycle.

Although the use of human endometrial epithelial cell lines such as HEC-1-A, RL95-2, and Ishikawa is well established in in vitro studies, it should be emphasized that these cell lines are derived from endometrial adenocarcinomas and may exhibit abnormalities in polarity regulation and cellular pathways. This is a limitation of such studies. Until now, no approved primary endometrial epithelial cell line has been available. Additional experiments should be performed with primary human endometrial epithelial cells which require clinical collaboration and approval by Ethics Committees. On the other hand, the cyclic changes of adhesion complex distribution on fixed human tissue sections (Figure 6, Figure 7 and Figure 8) support the translational validity of the results obtained in vitro with the endometrial cell lines.

Multiple studies [28,80,81,89,99,100] provided evidence that less polarized epithelial cells facilitate the invasion of the trophoblast.

PMT during embryo implantation has been compared to epithelial-to-mesenchymal transition (EMT), which is involved in morphogenesis, wound healing and cancer metastasis [102,103,104] and may also play an important role in endometriosis [103]. A systematic comparison between PMT and EMT has been provided by Whitby et al., 2020 [38]. During both processes there is a downregulation and/or rearrangement of adherens junctions, loss of apical microvilli, actin cytoskeleton rearrangements and loss of typical apicobasal polarity reflected by altered Scribble and Crumbs expression. Also, the co-occurrence of the intermediate filament protein vimentin, which is typical for cells of mesenchymal origin, and the cadherin switch from E-cadherin to N-cadherin in the poorly polarized endometrial cell line RL95-2 are signs of partial EMT. The higher level of NCad expression after hormonal stimulation with E2/MPA can therefore be taken as an indication of an epithelial-to-mesenchymal switch. However, there are certain differences between EMT and PMT as pointed out by Whitby et al., 2020 [38], especially regarding the disassembly of tight junctions, which is typical for EMT in cancer development but is not observed in the PMT in human endometrium during the menstrual cycle. Given these differences it is better to describe PMT as a process that includes some elements of EMT, mainly the reduction in the extent of cell polarity.

Several mechanisms are currently discussed that may affect endometrial receptivity by disturbed cell polarity and adhesion. Previous studies had revealed that microRNAs (miRs), which are known to affect endometrial receptivity through post-transcriptional gene expression, are dysregulated in the receptive endometrium of women with infertility [105,106]. A recent study by Zhou et al. [107] showed that microRNA 124-3p disturbed cell polarity and adhesion. The authors reported that miR-124-3p is overexpressed in endometrial luminal epithelium of infertile women during the receptive phase. Interestingly, microRNA-124-3p overexpression in primary human endometrial epithelial cells impaired embryo trophectoderm attachment in a 3D culture model. Elevated micro-RNA-124-3p in both mice and humans disrupted endometrial epithelial cell adhesion and polarity, preventing the uterine epithelium from becoming receptive [107].

Heng et al., 2021 [99] observed that overexpression of the transmembrane protein podocalyxin (PCX), which belongs to the CD34 family of sialomucins, leads to an upregulated expression of adherens and tight junction proteins in human endometrial epithelial cells. Paule et al., 2021 [100], identified PCX as a key negative regulator of human endometrial epithelial receptivity. PCX is strongly expressed in luminal epithelium during the proliferative, non-adhesive phase and downregulated in the mid-secretory phase. Experimental data with Ishikawa cells stably overexpressing PCX confirmed that it promotes epithelial barrier function by increasing the epithelial junction proteins E-cadherin and claudin 4. The increase in adhesion junctions prevents the switch to a less polarized receptive endometrial epithelium. PCX overexpression also increased epithelial endometrial cell polarity as determined by enhanced immunofluorescence staining of the polarity-determinant protein Scribble. Consistently, PCX overexpression decreased the attachment rate and speed of transepithelial invasion of trophoblast-derived spheroids [99,100]. It could be shown that PCX acts as a key orchestrator of apical cell morphology and can directly influence the recruitment of filamentous actin and ezrin to the plasma membrane inducing microvillus formation [108], which is typical for strongly polarized epithelia [99].

Previous studies suggested that the large membrane glycoprotein MUC-1 may also act as an antiadhesive molecule that must locally be removed by the human blastocyst during the adhesion phase [109]. However, in humans MUC-1 is present at the apical membrane surface throughout the menstrual cycle and even increases during the receptive phase [110].

Recently, Ruane et al., 2024 [111], found that glucose can influence endometrial epithelial cell receptivity by regulating posttranslational modification of proteins involved in the maintenance of cell polarity. They used the Ishikawa cell line in coculture with mouse embryos under different levels of glucose. High levels of glucose promoted blastocyst attachment by a reduction in epithelial cell polarity. That was caused by modulation of apical junctional function by GlcNAcylation of proteins in the ras homologous (Rho)/Rho-associated protein kinase (ROCK)/myosin light-chain signaling pathway.

## 5. Conclusions

In conclusion, morphological and functional studies listed and discussed in this review support the concept of endometrial epithelial cell polarity whereby strong epithelial cell polarity acts as a barrier and its transformation into a less polarized epithelium facilitates successful implantation.

Future research activities should focus on transepithelial trophoblast invasion, the very moment when the trophoblast crosses the epithelial layer and the specific cell–cell interactions, since the knowledge base for this part of the process of implantation is still rather rudimentary [97]. Only the studies of John et al. [72], Bentin-Ley et al. [54] and Ruane et al. [98] have addressed this specific process until now. Using spheroids created from stem cells [98] or primary human trophoblast cells [100], as well as artificial self-assembling embryo-like structures [66,112], three-dimensional endometrial explants [93] and endometrial organoids [113,114], will improve the currently available in vitro models to imitate the in vivo situation.

Finally, more elaborated in vitro models cells should include stromal cells, which have a strong impact on hormonal and paracrine regulation of epithelial cells [115,116,117,118,119,120] and play an important role in achieving a receptive state of the uterine epithelium.

## Figures and Tables

**Table 1:** Summary of different characteristics of human epithelial adenoarcinoma cell lines.

	HEC-1-A	Ishikawa	RL95-2	References
**Epithelial cell polarity**	high	moderate	low	[70,71,73,88,89]
**Adhesiveness**	non-adhesive	adhesive	highly adhesive	[70,73,89]
Keratin 8	**++**	**+**	(+)	[70,85]
Vimentin	Ø	single cells (+)	some cells (+)	[70,85]
E-cadherin	++	+	(+)	[70,85]
N-cadherin	Ø	Ø	(+)	[85]
Desmoplakin 1/2	++	+	+	[79,85,88,89]
ZO1	++	+	Ø	[79,85,88,89]

++ = strong; + = moderate; (+) = weak; Ø = negative.

## Data Availability

No new data were created or analyzed in this study. Data sharing is not applicable to this article.
